# Digital Literacy During COVID-19 Distance Education; Evaluation of Communication-Based Problems in Line With Student Opinions

**DOI:** 10.3389/fpsyg.2022.809171

**Published:** 2022-07-15

**Authors:** Sinem Kasımoğlu, Nesrin M. Bahçelerli, Mustafa Ufuk Çelik

**Affiliations:** ^1^Department of Communication, Near East University, Nicosia, Cyprus; ^2^Department of Tourism and Hotel Management, Near East University, Nicosia, Cyprus

**Keywords:** COVID-19, digital literacy, distance education, communication, student

## Introduction

The COVID-19 pandemic, which started in the People’s Republic of China in late 2019 and spread across the world at the beginning of 2020, has affected the health sector as well as other areas of activity worldwide (United Nations Educational Scientific Cultural Organization[UNESCO], 2020). The field of education has also been one of the areas that has been rapidly and significantly affected by the pandemic. Education and training activities carried out in all countries of the world within a certain plan and system for many years have had to undergo methodical changes in this process, and with the support of developing technology, education, and training activities at all levels from kindergarten to higher education have been moved to the computer environment (Zhong, 2020).

In times when technology was not sufficiently advanced, distance education was performed *via* mail and on demand; however, it has now moved to the internet environment with the support of rapidly developing technology, and teacher–student communication has started to take place through various technological devices, which have become mandatory during this period. Distance education processes, which have been frequently seen and experienced during the pandemic period, require a certain level of access to technology in today’s conditions and the ability to use this technology correctly, known as digital literacy (Lankshear and Knobel, 2015; Metin Taş, 2019).

The history of the phenomenon of “distance education,” which refers to situations where the education is carried out by the teacher and the student in separate physical environments (Akdemir, 2011), dates back to the eighteenth century; it is known that the announcement of stenography courses through distance education published in the Boston Gazette in 1728 was the first recorded example of distance education (Kırık, 2014). The distance education process, which began in the form of education by letter, continued with radio, television, teleconferences, cassettes, faxes, video conferences, and finally, the internet (Karagöz, 2019). It is seen that education that started in the computer environment over the internet has now transitioned to mobile devices with the development of mobile communication technologies (Yamamoto and Altun, 2020). The teaching style, which used to be defined only as “teaching by letter,” has now started to be characterized by different names such as “learning at home,” “open education,” and “distance education” (Aşkar and Altun, 2006). In the cases where the time allocated for education is limited, distance education is a teaching approach that allows the education process to continue (Tekedere, 2014). Distance education also helps to realize equal opportunities in education by enabling a standardized but tailored education to a wider audience at a lower cost without time and space restrictions (Telli, 2018).

The concept of distance education first appeared in Turkey between 1924 and 1927 and subsequently began to develop (Alkan, 1997). Letter teaching institutions and the open education system are the first examples of distance education in Turkey. During the pandemic period, formal education in Turkey and Northern Cyprus, as in the whole world, was moved to the Internet and television. This situation required the availability of the aforementioned electronic devices and internet access, as well as students, parents, and teachers having “digital literacy” skills.

“Digital literacy” can be defined as the ability to recognize, use, manage, analyze, and synthesize digital resources and tools, create new information, and communicate with others in this way (Martin, 2005), in addition to being able to use different information and communication technologies in daily life and educational processes. It refers to a wide set of skills including the functions of being able to access, produce, and share accurate information in the virtual environment (Hamutoğlu et al., 2017). According to Eshet (2002), digital literacy is a special and different way of thinking and not only includes the ability to obtain information over the internet but also the capacity to choose from and evaluated the information found. According to Gilster (1997), who first defined the concept of digital literacy, it is a special way of thinking, which not only includes pressing keys but also mastering the concept of the digital environment. It is not considered a new version or alternative to traditional literacy; in order to exist in today’s world in the norms of work, education, learning, and social life, it is a phenomenon that complements traditional literacy (Churchill et al., 2008).

Researchers have evaluated both the positive and negative aspects of the contributions of digitalization to the education and training processes, which have become particularly important to exist in today’s world. According to the researchers, the inclusion of digital technologies in education increases socialization and collaborative work (Baker, 2000), and provides rapid access to pre-written ready-made information and material on all the subjects *via* the Internet. However, the rapid access to information leads distracts them from creativity causing individuals to be lazy as everything is prepared for them (Evering and Moorman, 2012). The reliability of the information in the digital environment (Baker, 2000) and the increased likelihood of plagiarism (Scanlon and Neumann, 2002) are also among the negatives that may be caused by the digital environment. It is noted that these negativities may be due to the nature of technology and another important reason is the lack of digital literacy skills (Onursoy, 2018). It is seen that it is important to develop digital literacy skills in order to properly benefit from digitalization. Student–teacher communication in the education process comes to the forefront in the transformation of digitalization in the education process into positive and useful digital literacy skills.

Communication processes play an active role in the transition of the individual born and raised in society from a biological existence to a social existence (Tuna, 2014). The concept of communication, which can be defined as the process of sharing knowledge, thoughts, feelings, attitudes, and skills (Oğuzkan, 2003) between a message source and a target in order to change behavior, is viewed from the point of view of the education process; the interaction process between the student and the teacher, which takes place within a certain curriculum and where the purpose of changing behavior is at the forefront. While teacher–student communication is also face-to-face in traditional education, in distance education, this communication takes place through technological tools such as computers, televisions, telephones, and teleconference systems. This can lead to different communication problems than in face-to-face training.

Elcil and Sözen-Şahiner (2014) listed the barriers to the distance education process as being disruptive, constructive, channel-sourced, personal, physical, technical, psychological, temporal, student-induced, and educator-induced barriers. Student communication problems include situations such as the student’s reluctance to communicate with the faculty member, the inability to ask questions, or the lack of interest in the course. Educator communication problems include late responses to the students’ questions or no answers at all, unfair behavior when evaluating students, and excessive authoritarian attitudes. All these elements have various levels of impact on student and teacher communication in the distance education process.

In this process, where the structure and nature of the distance education process are different from face-to-face education, it is clear that the communication between the student and the teacher will develop in different forms from the face-to-face training process. It is thought that the digital literacy skills of the students in this communication process will affect the communication process. The main purpose of this study is to examine and demonstrate the digital literacy skills of the students in the distance education process, which has become not a choice but a necessity for the whole world as a result of the pandemic.

## Method

In the research, the case study technique, which is one of the qualitative research methods, was used. The research data were collected through a structured interview form prepared by the researchers. While preparing the research questions, the necessary corrections were made by taking the opinions of two academicians who are experts in the field. The research data were collected through digital platforms. The study group consisted of 23 students from the Near East University Faculty of Communication, Radio, Television, and Cinema Department. Participants were determined by the purposeful sampling technique. Participation in the research was carried out on a voluntary basis. It was stated to the participants that their identifiable information would be kept confidential and would only be used within the scope of the research. The content analysis method was used in the interpretation of the data. The data obtained by the content analysis were classified by coding and the same topics were collected under similar themes. Participant opinions are given in quotation marks and italics. In order to ensure the anonymity of the participants, each participant was identified as S1, S2, etc. Table 1 showed that the demographic characteristics of the participants.

**TABLE 1:** Demographic and communication tools used distribution of participants.

	*N*%	%
**Gender**		
Female	6	26
Male	17	74
**Age**		
19–24 years	16	70
25–29 years	5	21
30 years and older	2	9
**Education level**		
Undergraduate	22	96
Master		4
**Distance education accessing format**		
Mobile phone	6	27
Tablet	1	5
Personal PC	15	68

## Findings and Discussion

The research findings are shown in Table 2. The research participants were asked for what purpose they used digital education and online platform applications communicatively during the pandemic process. The opinions of the participants included for the purpose of communicating with family, friends, and teachers (n21), following the country and world agenda (n16), learning the latest information about the disease (n12), following the developments related to my interest/department in which I am studying (n12), chat and entertainment purposes (n11), watching movies, listening to music, etc. (n11), participating in discussions and commenting (n4), and spending time (n6). When the responses of the participants were examined, it was found that they used digital education and online platform applications to communicate with their family, friends, and teachers the most.

**TABLE 2:** Digital literacy in distance education given during COVID-19; student opinions on communication-based problems.

Theme	Sub-theme		*n*	%
Digital education and the use of online	To communicate with my family, friends and teachers		21	23
platform applications for communication	To follow the country and world agenda		16	17
purposes during the pandemic				
	To keep track of developments related to my interest/department in which I am studying		12	13
	For gaming, chat, entertainment purposes		11	12
	For the purposes of watching movies, listening to music, etc.		11	12
	To participate and comment in discussion		4	4
	To pass the time		6	6
Positive and/or negative factors with	Positive	Before, face-to-face training was more effective.	6	38
regard to communication in the process of		Student-centered education	7	44
education, compared to the pre-pandemic		Communication increased	1	6
period		Opportunity to study and follow courses	2	12
	Negative	Increased of assignments/projects	4	13
		Difficulty to communicate	9	29
		Lack of hands-on lessons	1	3
		Lack of motivation/uncertainty	3	10
		Technological infrastructure	3	10
		Attendance at the class has become difficult	5	26
Communication with family, friends and the	To be able to communicate with everyone at any time		14	34
environment in the pre-pandemic period	Participate in family/friend events		15	37
	We used to communicate more often.		12	29
Differences in communication processes	Not being able to meet		10	25
with family, friends and other people during	Not organizing/participating in events		8	20
the pandemic	We started seeing each other at home.		4	10
	Social distancing and hygiene rules were effective		6	15
	Increased conversations online		12	30
Positive and negative factors with regard	Positive	Not to see each other so as not to spread the disease	2	8
to communication process compared to		Communication was warmer before	6	25
the pre-pandemic period		Not much has changed	4	17
		Environmental awareness has increased	2	8
		Compliance with the rules has increased	6	25
		Communication has become more frequent	4	17
	Negative	Psychological difficulties	6	38
		Internet addiction has increased	5	31
		Communication lost	3	19
		Financial difficulties	2	13
Recommendations for the post-pandemic	Studies should be carried out in this direction		1	5
remote communication process	It causes people to be distanced		3	15
	Infrastructure must be improved		7	35
	Transition to face-to-face training must be ensured		5	25
	Empathy must be developed		2	10
	Crisis management training should be provided		2	10
Effects of digital education and online	Interest in digital education and online platforms has increased		7	17
platform elements on communication	Internet and social media are being actively used		14	34
processes between people	Negative psychological effects have emerged		2	5
	Technological dependence increased		11	27
	Success is achieved		2	5
	Information pollution has increased		4	10
	Communication is weakened		1	3

When the participants compared the pre- and post-pandemic period, the participants were asked what the positive and/or negative elements were with respect to communication in the process of education, and the positive responses of the participants were collected under 4 sub-themes and their negative responses were collected under 6 sub-themes. The positive opinions of the participants are as follows: face-to-face education was more effective before the pandemic (n6), student-centered education (n7), increased communication (n1), and opportunity to study and follow courses (n2). Negative opinions were: increased homework/projects (n4), difficulty in communicating (n9), lack of hands-on courses (n1), lack of motivation/uncertainty (n3), technological infrastructure (n3), and difficulty in communicating (n5). The expressions of the participants in this direction are as follows:

*”I don’t think there are any positives. The negative was not being motivated, and not being able to ask my teachers questions if I find the time and watch it again.” S3*

*”With the increase in the assignments and projects that I see as positive, we are directed toward research. Negatively, communication and the atmosphere of the course are not like in the classroom”* S11

The participants were asked about their communication patterns with family, friends, and the environment in the pre-pandemic period, and their responses were collected in 3 sub-themes. These were the ability to meet with everyone at any time (n14), to attend family/friend events (n15), and to communicate more frequently (n12). The opinions expressed by the participants in this direction are as follows:

*”I was in touch with so many friends and we would meet all the time. After the pandemic started, we did not have the opportunity to meet with anyone face-to-face, communication decreased.”* S23

*”Since I study abroad, my parents and I were on the phone and it didn’t change much. According to the degree of closeness with my friends and other people, we used to do activities in places such as visiting each other’s homes, going to cafes, cinemas and restaurants.”* S3

In continuation of this question, participants were asked about the differences in the communication processes with family, friends, and the environment during the pandemic. Participants expressed the views of not meeting people (n10), not organizing/participating in activities (n8), we started seeing each other at home (n4), social distancing and hygiene rules were effective (n6), and online meetings increased (n12). Some participant’s statements included:

*”After the pandemic started, some of my friends returned to their hometowns. I stayed in Cyprus with a small number of my friends. Although it is not a social activity, we started meeting at home.”* S5

*”Communication only happens through social networks. That’s the number of people I meet online. We video chat every day*.” S7

When the pre- and post-pandemic periods were compared, it was asked what the positive and negative elements were in terms of communication. Positive expressions of the participants in this direction were collected under 6 sub-themes, while negative expressions were collected in 4 sub-themes. Positive statements included: communication was warmer before (n2), communication was more frequent (n4), environmental sensitivity increased (n2), compliance with the rules increased (n6), and communication became more frequent (n4). On the other hand, negative statements included: psychological difficulties (n6), increased internet addiction (n5), communication lost (n3), and financial difficulties (n2). The statements made by the participants included:

”*The positive side of this process is that we don’t meet people and spread the disease any more. The downside is that we are going through psychologically difficult period because we have not met with anyone*.” S1

The recommendations put forward by the participants for the remote communication process after the pandemic are as follows; studies should be carried out in this direction (n1), people were distanced (n3), infrastructure should be improved (n7), face-to-face education should be transitioned (n5), empathy should be developed (n2), and crisis management training should be given (n2). The participants’ recommendations are as follows:

*”Many people had problems with the introduction of distance education during the pandemic. Unfortunately, both the reasons for the infrastructure problems in the country and the lack of access to distance education have damaged the right of individuals to receive equal education. I think that the infrastructure should be improved and every student should have access to the courses.” S9* Ramos-Morcillo et al. (2020) emphasized that one of the downsides of distance education is that students do not have equal rights.

*”I think face-to-face education is more efficient and effective. There must be ways to transition to face-to-face education.”* S21

The participants were asked what the effects of digital education and online platform elements were on communication processes between people. The participants stated these effects included increased interest in digital education and online platforms (n7), internet and social media started to be actively used (n14), negative psychological effects appeared (n2), technological dependence increased (n11), success was achieved (n2), information pollution appeared (n4), and communication was weakened (n1). Some participants stated:

*”With the move of education to digital platforms, people started to use the internet and social media more. Dependence on technology has increased.” S13*

*”The use of technology has increased a lot and everyone has had something to say about the events that have happened, and so information pollution has emerged. What information is correct is being questioned.” S18*

## Conclusion, Discussion, and Recommendations

During the COVID-19 pandemic, evaluations were made in line with student opinions in order to convert the students’ thoughts and processes into positive in the problems based on distance education digital literacy, and communication. In addition to the finding that the student expectations in the education process have not adequately been met, the level of digital literacy is also considered inadequate to understand the educational infrastructures. Many studies in the literature have investigated the effects of COVID-19 on education. As a result of the researchers’ studies, it has been revealed that communication is one of the leading problems in this process for students and lecturers (Basilaia and Kvavadze, 2020; Dhawan, 2020; Petretto et al., 2020; Huang et al., 2020). The research shows parallelism with the literature in line with the findings.

It is also seen from the results obtained from the student opinions that the absence of students from the regional location, economic difficulties, and comfort also play an important and negative role in educational saturation. Among the findings of the study, the phenomenon of miscommunication in the learning process of individuals and feelings of isolation and inadequacy were highlighted. Studies have supported these findings. It is stated that during this process, learners experience communication problems while trying to adapt to the new educational environment (Can, 2020; Daniel, 2020; Telli and ve Altun, 2020). In addition to all these negative findings, the positive element is also indicated as the possibility of replaying the trainings irrespective of time or location in line with student opinions on digital platforms.

One of the most important indicators of this process is that there are infrastructure and communication problems. It is recommended to introduce planning and development practices in order to follow new technologies in distance education and to provide equal opportunities to students in this direction. It is recommended to adapt distance education technologies to education programs. Seminars and in-service training programs for distance education and technologies can be organized for all the stakeholders of education. This research was carried out with the participation of university students. The same research can be repeated with the participation of primary, secondary, and high school students and the findings can be compared.

## Data Availability Statement

The original contributions presented in the study are included in the article/supplementary files, further inquiries can be directed to the corresponding author/s.

## Ethics Statement

The studies involving human participants were reviewed and approved by Near East Ethical Committee Board. The patients/participants provided their written informed consent to participate in this study.

## Author Contributions

All authors listed have made a substantial, direct, and intellectual contribution to the work, and approved it for publication.

## Conflict of Interest

The authors declare that the research was conducted in the absence of any commercial or financial relationships that could be construed as a potential conflict of interest.

## Publisher’s Note

All claims expressed in this article are solely those of the authors and do not necessarily represent those of their affiliated organizations, or those of the publisher, the editors and the reviewers. Any product that may be evaluated in this article, or claim that may be made by its manufacturer, is not guaranteed or endorsed by the publisher.
